# Performance Comparison of a Neural Network and a Regression Linear Model for Predictive Maintenance in Dialysis Machine Components

**DOI:** 10.3390/bioengineering12090941

**Published:** 2025-08-30

**Authors:** Alessia Nicosia, Nunzio Cancilla, Michele Passerini, Francesca Sau, Ilenia Tinnirello, Andrea Cipollina

**Affiliations:** 1 Dipartimento di Ingegneria, Università degli Studi di Palermo, Viale delle Scienze Ed. 6, 90128 Palermo, Italy; alessia.nicosia@unipa.it (A.N.); ilenia.tinnirello@unipa.it (I.T.); andrea.cipollina@unipa.it (A.C.); 2 Mozarc Medical^®^, Via Camurana 1, 41037 Mirandola (MO), Italy; michele.passerini@mozarcmedical.com (M.P.); francesca.sau@mozarcmedical.com (F.S.)

**Keywords:** hemodialysis, machine learning, LSTM, prediction

## Abstract

Ensuring the reliability of dialysis machines and their components, such as sensors and actuators, is critical for maintaining continuous and safe dialysis treatment for patients with chronic kidney disease. This study investigates the application of Artificial Intelligence for detecting drift in dialysis machine components by comparing a Long Short-Term Memory (LSTM) neural network with a traditional linear regression model. Both models were trained to learn normal patterns from time-dependent signals monitoring the performance of specific components of a dialytic machine, such as a weight loss sensor in the present case, enabling the detection of anomalies related to sensor degradation or failure. Real-world data from multiple clinical cases were used to validate the approach. The LSTM model achieved high reconstruction accuracy on normal signals (most errors < 0.02, maximum ≈ 0.08), and successfully detected anomalies exceeding this threshold in complaint cases, where the model anticipated failures up to five days in advance. On the contrary, the linear regression model was limited to detecting only major deviations. These findings highlighted the advantages of AI-based methods in equipment monitoring, minimizing unplanned downtime, and supporting preventive maintenance strategies within dialysis care. Future work will focus on integrating this model into both clinical and home dialysis settings, aiming to develop a scalable, adaptable, and generalizable solution capable of operating effectively across various conditions.

## 1. Introduction

In recent years, Artificial Intelligence (AI) has become one of the most impactful and transformative tools across various sectors, including business [1], tourism [2], telecommunications [3], and healthcare [4]. Its rising influence stems from its capability to analyze complex data, respond dynamically to changing conditions, and automate decision-making processes with a degree of precision and reliability that traditional systems have been unable to achieve.

AI utilizes sophisticated mathematical systems to carry out its functions [5]. Some systems operate based on simple rules and logic, while others use more advanced algorithms that enable them to learn and adapt over time. Under the common name of “Artificial Intelligence” several subfields exist [6], with Machine Learning (ML) emerging as one of the most prominent in recent years. ML is a branch of AI that focuses on enabling computers to learn from data and enhance their performance over time without being explicitly programmed. The foundation of machine learning lies in what is known as “training”. To train an ML model, it must be provided with a dataset that includes examples relevant to the problem it is meant to solve. Once trained, the model can then generate quick and accurate predictions on new, unseen data, referred to as “testing data”.

AI in healthcare was first explored in the 1960s, notably for electrocardiogram (ECG) interpretation. Although the potential of AI was already recognized at the time, its advancement remained relatively stagnant for more than a decade. A key advance came when Gagliardo [7] applied multilinear regression to predict tumor antigens in melanoma patients, using a dataset analyzed by Heppner et al. [8] in 1973 through classical statistical methods. This marked the beginning of a sustained and expanding use of AI in biomedical research, including applications in chronic kidney disease and renal replacement therapies, a topic to which this article aims to contribute. Chronic kidney disease (CKD) is a global health burden affecting millions of individuals worldwide [9], often progressing to end-stage renal disease (ESRD), a pathology in which blood flow to the kidneys is reduced due to renal artery disease and where renal replacement therapies, such as hemodialysis (HD) [10], become essential for patient survival. This treatment is carried out using hemodialyzers, installed within dialysis machines, which may range from large, sophisticated hospital systems operated by medical staff, to smaller, portable units intended for home use by patients with residual kidney function [11].

In recent years, the use of artificial intelligence models in the field of dialysis has primarily focused on creating predictive and non-invasive solutions to address complications that may arise during or after treatment [12]. The aim is to minimize the risk of further health deterioration in patients who are already in a compromised condition. Issues such as unstable fluid volumes or blood pressure during dialysis sessions, as well as treatment-related complications like muscle cramps or damage to the arteriovenous fistula (AVF), represent significant challenges for patients undergoing dialysis. In this regard, Ota et al. [13] developed an innovative AI model designed to analyze the acoustic signals produced by the arteriovenous fistula in order to detect stenosis, a condition caused by narrowing of the fistulized blood vessel. Similarly, Balamuthusamy et al. [14] explored the use of ML models developed on a Microsoft Azure cloud computing platform for prediction of re-intervention risks in patients with arteriovenous (AV) access. Employing a range of methods, from basic statistical techniques to advanced machine learning models, numerous researchers have concentrated on predicting complications such as muscle cramps [15,16] and intradialytic hypotension (IDH) [17,18] in patients undergoing dialysis [19]. Interesting is also the development of AI models aimed at studying fluid overload in hemodialysis patients as done by Tan et al. [20], who implemented a model to evaluate the fluid status of patients, since significant fluid overload is often responsible for various lung pathologies (e.g., pulmonary edema). In 2018, Niel et al. [21] employed machine learning techniques to enhance the accuracy of dry weight estimation, the target amount of fluid to be removed during a dialysis session to maintain proper fluid balance in the body. Another significant application of AI in the field of dialysis is the prediction of long-term therapy outcomes, which may span days to months or even years in advance. The goal is to assess patients’ clinical conditions and anticipate potential complications, including mortality. Among the researchers addressing this challenge, Díez-Sanmartín et al. [22] developed an AI model aimed at forecasting mortality in dialysis patients based on their health status and sociodemographic characteristics.

Other studies have specifically focused on dialysis equipment and its critical role in managing the entire dialysis process, particularly in predicting machine-generated alarms. All dialysis machines share a common feature: the presence of numerous sensors and pumps that regulate and monitor the process. Key factors such as blood pressure, minimal protein loss and efficient removal of urea (the main marker for the dialysis performance) are in fact monitored during the treatment [10]. Nevertheless, several risks may arise during treatment, including technical malfunctions and changes in the patient’s clinical condition. To address these risks, modern dialysis machines are equipped with alarm systems designed to alert the operator and, if necessary, halt the procedure. These alarms are activated in response to abnormalities such as deviations in blood pressure, temperature, heart rate, blood loss, or issues related to the machine’s ultrafiltration, degassing, or fluid delivery systems. However, these systems typically respond only after a problem has occurred or is imminent, limiting the time available for medical personnel to intervene and prevent the situation from deteriorating or the machine from shutting down.

In this context, a previous study from the same authors [23] focused on developing predictive models based on machine learning algorithms to anticipate the occurrence of alarms during the patient treatment phase in dialysis. These alarms, which are triggered by imminent abnormal patient conditions, such as deviations in blood pressure, temperature, or other physiological parameters, are generated by the machine’s safety systems and do not indicate technical malfunctions. Several predictive models were evaluated, with the Random Forest classifier proving to be the most effective, achieving an F_1_ score of up to 80% in predicting alarms 10 s prior to their activation. These findings highlight the potential of AI to improve clinical alarm management by providing medical staff with additional time to intervene proactively, thereby significantly enhancing patient safety during dialysis treatment. Similarly, in other non-dialytic equipment, Raboshchuk et al. [24] developed an AI model to detect alarm sounds from biomedical devices in a neonatal intensive care unit. Their model aimed to enhance alarm detection in noisy environments, targeting both technical and clinical alarms, including out-of-range blood pressure, oxygenation levels, and abnormal heart rates. The system demonstrated significant improvements in alarm accuracy, reducing false positives up to 70% compared to traditional systems and thereby enhancing reliability in critical care settings.

While much of the literature has focused on predicting complications directly related to patient conditions, relatively few studies have examined sensor-related alarms in complex medical devices. In particular, there is a significant research gap regarding the development of models capable of forecasting sensor failures outside the treatment phase, such as predicting the outcomes of pre-use diagnostic tests conducted to verify measurement accuracy. This issue is critical, as these procedures can be time-consuming and may delay the activation or availability of the devices housing the sensors. Moreover, even during active operation, devices frequently experience intermittent interruptions caused by repeated sensor tests intended to verify ongoing reliability. Predictive models that utilize the complete operational history of individual sensors, monitoring their performance from installation onward, could significantly reduce the need for such tests, thereby minimizing system downtime and interruptions. In some cases, these models may prove more reliable than the tests themselves by using longitudinal data to identify subtle trends indicative of sensor degradation or impending failure. Ultimately, these predictive approaches could enhance operational efficiency, shorten diagnostic and verification cycles, and support the continuous, uninterrupted use of both sensors and the overall system.

In this regard, one of the few notable efforts addressing this issue is the work of Rahman et al. [25], who proposed a predictive maintenance AI model for critical medical devices aimed at optimizing utilization and minimizing downtime. Their model analyzed data from 8294 critical medical devices across 15 hospitals in Malaysia, encompassing multiparametric monitors, ventilators, diagnostic imaging equipment, life support systems, and infusion pumps. The study assessed mechanical, electronic, calibration, system, and power failures, predicting the probability of device failure within three years with an accuracy of up to 79.50%, which is a significant achievement given the diversity and complexity of the devices and their potential failure modes.

**Figure 1:**
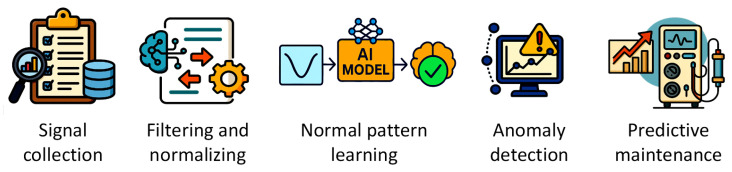
Research workflow for predictive maintenance of dialysis machine sensors, from data collection to AI-based anomaly detection and early-warning support.

Building on similar thematic and modelling approaches, this study focuses on predictive maintenance for a specific medical device. It presents a framework that compares the performance of an LSTM neural network with a traditional linear regression model for detecting and monitoring anomalies in critical components of dialysis machines, several days before alarms are triggered. Specifically, it analyzes the behavior of the sensor that measures patient weight loss during treatment, aiming to anticipate abnormal patterns that may indicate equipment breakdowns requiring technical intervention. The research workflow, summarized in Figure 1, illustrates the main steps of the study.

Among the various sensors integrated into the dialysis machine, the weight loss sensor plays a particularly critical role. Its accuracy directly affects the estimation of fluid removal during treatment. Any issue can result in incorrect ultrafiltration volumes, putting the patient at risk of excessive or insufficient fluid loss, or even causing premature interruption of the dialysis session. For this reason, this component represents a priority target for predictive monitoring and is specifically addressed in this work.

## 2. Materials and Methods

### 2.1. Sensor and Context Description

Dialysis machines are equipped with an intricate internal hydraulic circuit that integrates various actuators and sensors responsible for regulating and monitoring critical treatment parameters. This closed-loop system ensures the precise control of fluid flow, pressure, temperature, and conductivity, which are critical factors for the safe and effective delivery of dialysis treatment. Over time, however, certain components within this circuit may undergo performance *drift*, defined as a gradual deviation in measurement accuracy caused by mechanical wear, biological exposure, or aging effects. In other cases, components may experience an outright *malfunction*, understood as a sudden or severe loss of functionality that prevents correct operation. Both conditions can compromise system reliability and patient safety, making regular calibration or, when necessary, component replacement essential. Clear monitoring of sensor drift and timely identification of malfunctions are therefore critical for maintaining optimal machine performance and preventing unexpected interruptions in treatment delivery [26].

In several commercial dialysis machines, the weight loss sensor undergoes automatic self-testing to ensure proper functionality and to prevent the session from continuing in the event of malfunction. This test checks the internal hydraulic pressures to detect potential sensor drift. When the sensor is functioning correctly, the pressure–time graph observed during the test exhibits a characteristic symmetrical “V-shape”.

Implementing a system capable of intelligently monitoring this pressure shape to detect potential drift in advance, before the machine issues an alarm requiring sensor calibration or replacement, is crucial.

### 2.2. Signals Time-Series Pre-Processing

Circuit pressure data collected during the weight loss sensor self-tests of a dialysis equipment were used to train and test the model. A total of ~150 pressure–time graphs were analyzed, all representing normal operating conditions without drifts or malfunctions. This dataset was intentionally limited to ideal cases to enable the model to effectively learn and generalize the expected pressure patterns during standard sensor self-tests. Following standard machine learning practice of adopting an 85-15 split of the available data [27], 103 signal time-series (~85%) were allocated for training, while the remaining 20 signals (~15%) were reserved for testing the model’s performance. Within the training set, 20% of the sequences were further held out as a validation set. This validation subset was used exclusively to monitor the model’s generalization performance during training and to guide hyperparameter tuning. Notably, this setup ensured that no information from the test set was used during model development, thus preventing data leakage and overfitting.

**Figure 2:**
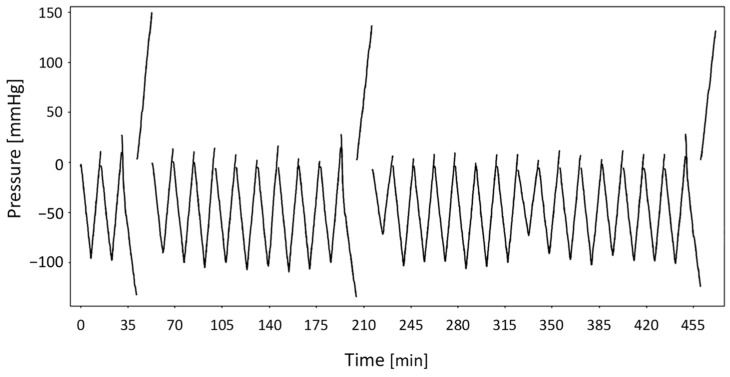
Pressure–time signals filtered to include only their actual phases.

Regarding the testing procedure of the dialysis machine, two types of sensor self-tests are performed. While both signals exhibit a similar shape, they differ in duration: the pre-treatment self-test lasts ~140 s and includes a transition phase characterized by a noticeable jump, that is absent from the periodic self-tests conducted every 30 min during the treatment, each of which lasts ~70 s. All signals are acquired at a sampling frequency of 2 Hz (two measurements per second). To improve learning quality and minimize noise, each signal time-series (hereafter indicated as “signals”) underwent a systematic pre-processing step. Specifically, for each longer signal, the rising branch was shifted to the minimum pressure point of the subsequent descending branch. This step was taken to exclude transitional phases that might interfere with the model’s ability to capture consistent trends. This feature provided a consistent temporal reference across recordings, without affecting the interpretation of the signals. The overall shape of these filtered pressure signals is shown in Figure 2.

Each signal was then normalized on an individual basis, according to the following formula:

(1) $$x_{norm}\left(t\right)=\frac{x\left(t\right)-x_{min}}{x_{max}-x_{min}}$$

where $x\left(t\right)$ is the raw pressure at time t, and $x_{min}$ and $x_{max}$ are the minimum and maximum values of the same signal, respectively. The normalization ensured that all signals were rescaled within the [0, 1] interval, making them directly comparable across tests, regardless of their absolute pressure values.

**Figure 3:**
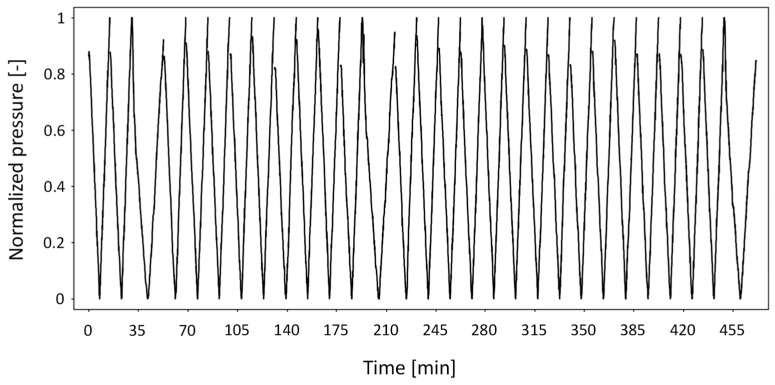
A subset of the dataset used to train the AI model. These signals correspond to those shown in Figure 2, after filtering and normalization.

After filtering and normalization, a total of 123 high-quality signals remained and were used for model training and testing. Specifically, 80 of the shorter tests and 23 of the longer tests were used for training, while 15 of the shorter and 5 of the longer tests were reserved for testing. This split preserved the typical minimum ratio of ~1:4 between long and short signals, as observed in a standard dialysis session lasting at least two and a half hours [28]. Furthermore, the relatively small number of tests was deemed sufficient for training, as the repetitive and regular nature of the signal shapes enabled the network to achieve good performance despite the limited dataset. After pre-processing, the final dataset consisted of signals with the shape illustrated in Figure 3.

### 2.3. LSTM Model

A Long Short-Term Memory (LSTM) network [29] was employed as the predictive model, implemented in Python 3.13 using TensorFlow 2.18 (Keras API v3.5), and run on a workstation equipped with an Intel^®^ Core™ i7-1185G7 CPU and 16 GB of DDR4-3200 RAM (Intel Corporation, Santa Clara, CA, USA). LSTM is a type of Recurrent Neural Network (RNN) specifically designed to learn from sequential data and capture long-term dependencies. Unlike traditional RNNs, which suffer from gradients that vanish or explode during training, LSTM networks can regulate the flow of information and maintain memory over long sequences. This capability makes LSTM particularly effective for tasks such as time-series prediction, where identifying patterns over time is crucial. In the context of reconstruction-based anomaly detection, LSTMs function as unsupervised neural networks, aiming to learn the normal behavior of a system from historical data and then identify deviations that might indicate anomalies. Within this framework, the LSTM learns to predict future values based on past observations, with poor prediction performance often indicating abnormal or unexpected behavior in the input data. As LSTM models process data sequentially, it is necessary to define the timesteps hyperparameter before training. This parameter specifies the number of past data points the model should consider simultaneously when making a prediction. In other words, timesteps define the length of the input sequence provided to the model at each stage of training. For this application, the number of timesteps was set to 250, enabling the LSTM to learn temporal dependencies on 250 data points and continuously predict the next value at each step during the entire sequence. This specific value was chosen to ensure that the sliding input window would always contain at least one complete pressure–time signal pattern. Regarding the architecture, the model comprised a single recurrent layer with 128 hidden units and a ReLU activation function, followed by a linear dense output layer. Training was conducted using the mean squared error (MSE) loss function, which was minimized using the Adam optimizer with a learning rate of 0.01, a batch size of 64, and running for up to 200 epochs. Although no explicit regularization techniques were applied, overfitting was mitigated through early stopping with a patience of 20 epochs, restoring the best-performing weights.

The present study is based on the principle that an LSTM model, once trained on normal data, can accurately reconstruct typical sequences, while anomalous patterns lead to higher reconstruction errors. It is worth noting that model evaluation and anomaly detection, defined as any significant deviation from the “clean” signal pattern potentially reflecting either sensor drift or outright malfunction, were based on the absolute reconstruction error [30], rather than the MSE used during training. This choice was made because MSE ensures stable convergence of the network during training, while the absolute error offers an intuitive, signal-level metric that can be directly compared against a fixed threshold to identify abnormal deviations. The absolute reconstruction error is formally expressed as:

(2) $$\left|x-\hat{x}\right|$$

where $\hat{x}$ is the predicted value and x is the actual value. Since the data were normalized to a range between 0 and 1, the resulting absolute errors also fell within this range.

**Figure 4:**
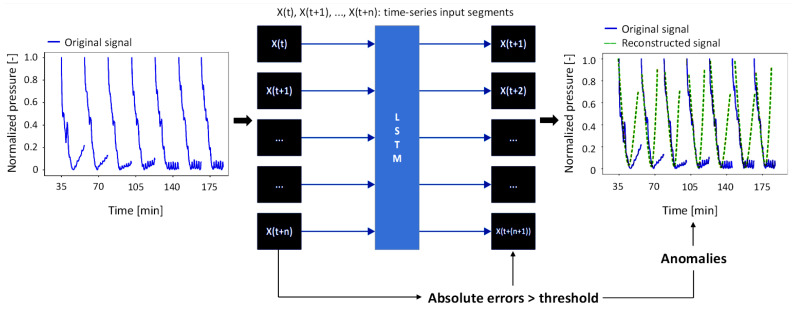
Working principle of the LSTM model used for anomalies detection. Legend: solid line (blue signals): original anomalous input; broken line (green signals): reconstructed “correct” version generated by the model; X(t), X(t + 1), …, X(t + n): time-series input segments used by the model to learn and predict future system behavior.

Figure 4 shows the working principle of the LSTM model in this application.

To ensure accurate training, it was necessary to calculate the absolute errors for all clean data samples and verify that they were close to 0. To detect anomalies in the analyzed sequence, the absolute errors for all samples were recalculated, and one of the predefined criteria was applied to determine which deviations in the signal reconstruction should be considered anomalies. The chosen criterion is based on threshold exceedance: after sorting all absolute errors and setting a threshold value, any point with an absolute error exceeding this threshold is classified as an anomaly.

After evaluating the model’s generalization capability, several trials were conducted using data containing anomalies. Anomalies were identified using a maximum threshold of 1, and various ranges of potential anomalies were defined for analysis. These ranges were then classified according to levels of significance and severity, based on the comparison between the anomalies identified by the model and those reported by technicians during the complaint investigation stage.

## 3. Results

**Figure 5:**
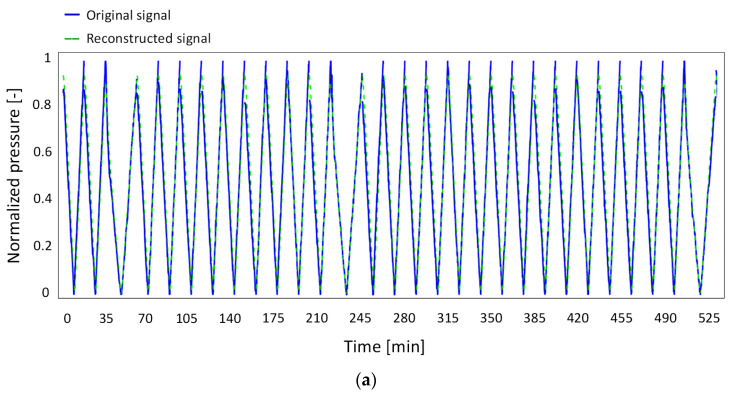

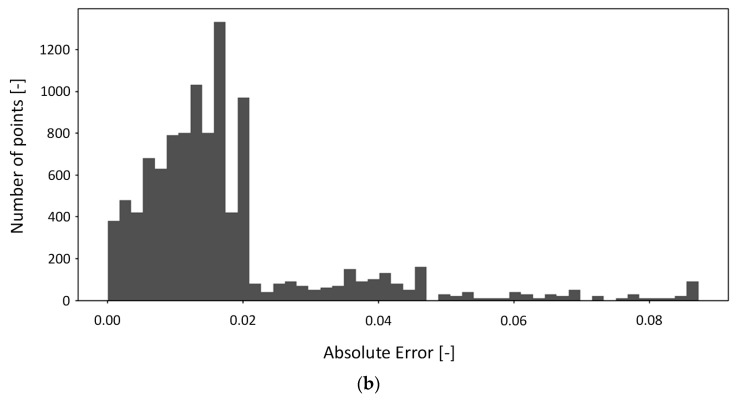
(**a**) Comparison between original signals (solid blue line) and network-reconstructed signals (dashed green line) during the training; (**b**) histogram showing the distribution of the absolute reconstruction errors in the training phase.

The results of the training phase are shown in Figure 5a, which reports the reconstructed signals generated by the network (dashed green line) and the original signals (solid blue line) as a function of time. Figure 5b shows, in the form of histogram, the distribution of the absolute reconstruction errors during the training phase. The signals were reconstructed with high accuracy, with a maximum reconstruction error of ~0.08 and the majority of the data points exhibiting an absolute error below 0.02.

Testing the model on the remaining ~15% of the entire dataset consistently yielded a maximum reconstruction error of ~0.08, following the same trend observed in the training set, as illustrated in Figure 5. This confirmed that the previously established threshold represents the model’s reconstruction capability limit, which cannot be further improved through additional training. This technical validation was further supported by comparing the model’s predictions with real-world complaint cases reported by hospitals. Anomalies associated with reconstruction errors exceeding 0.08 were consistently confirmed by technicians during post-event analyses of treatment-related drifts, while errors below this threshold were not indicative of actual drifts. Consequently, reconstruction errors larger than 0.08 were used as indicators of anomalies in signals that exhibited irregular behavior.

Specifically, twelve complaint cases were analyzed, divided into three different categories with four cases in each category. Each case included the reported incident along with approximately one month of previous treatment data. The objective was to evaluate the effectiveness of the model in confirming the problem reported by the operator and, more importantly, its ability to anticipate weight loss sensor self-test failures (i.e., unsuccessful self-test outcomes) early enough to allow for preventive intervention. Table 1 summarizes the three categories considered. The first column indicates the name assigned to each case, each corresponding to a different anomaly condition analyzed in this study.

In the following, two cases from each main category will be presented and discussed from Section 3.1, Section 3.2, Section 3.3 and Section 3.4, to ensure coverage of at least one case from each sub-category. In addition, Section 3.5 will include a comparative analysis between the LSTM model and a simple linear regression approach to assess their respective performance on the same dataset.

### 3.1. Machine out of Order Due to Weight Loss Sensor Self-Test Failure

#### 3.1.1. Failure Related to Weight Loss Sensor Drift

**Figure 6:**
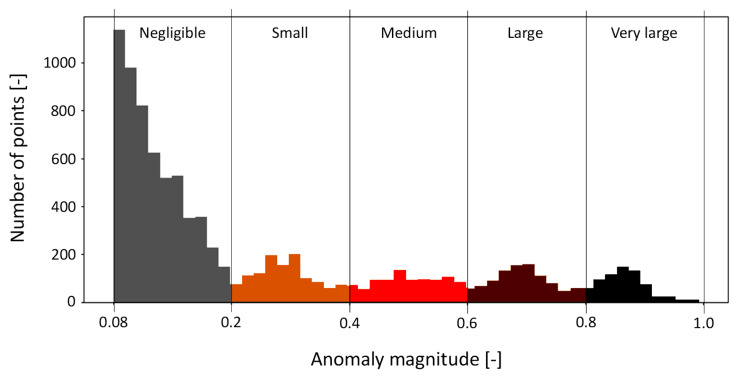
Histogram of anomalies distributed according to their magnitude. The description of the issue associated with each range is reported in Table 2.

Anomaly condition Type 1 involves a complaint regarding a machine being out of service due to the failure of a sensor self-test for the weight loss sensor. Despite an attempt to recalibrate the component after the initial failure, a second failure occurred a few days later. Figure 6 shows the magnitude of the anomalies detected during the reconstruction of the signals under investigation, which range from 0.08 to 1.

Five ranges of anomaly severity were defined, with each range assigned a different color to facilitate visual interpretation. Since the first range was the most prevalent, it was excluded from the following graph and the subsequent ones to better highlight the distribution of the remaining severity ranges.

**Figure 7:**
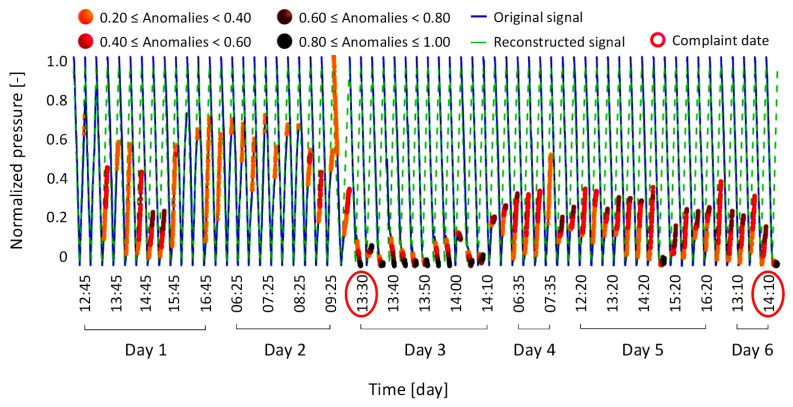
Results of the anomaly condition Type 1, where a drift in the weight loss sensor caused a machine failure.

**Figure 8:**
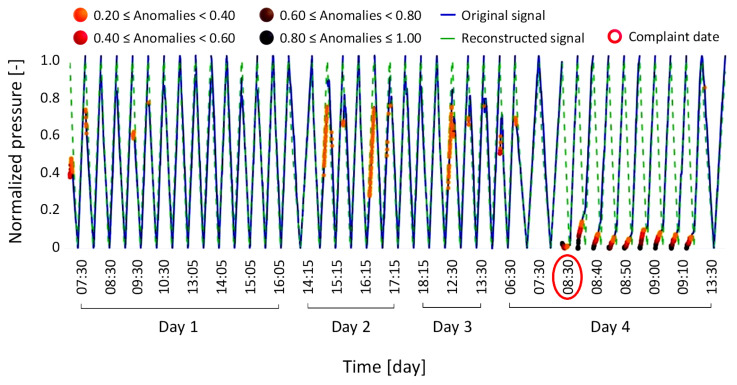
Results of the anomaly condition Type 2, where a solenoid valve rupture caused a machine failure.

**Figure 9:**
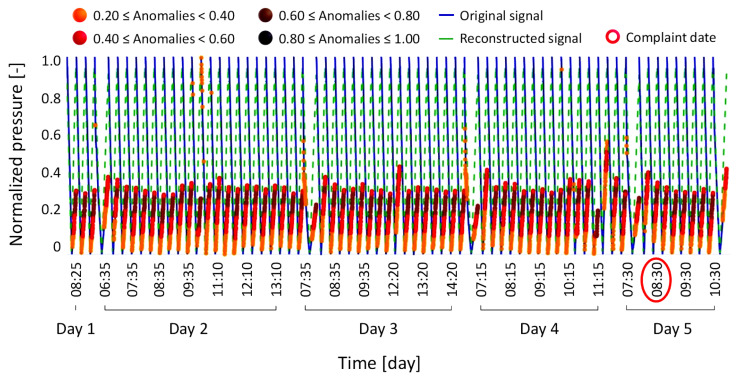
Results of the anomaly condition Type 3, where a drift in the weight loss sensor did not result in a machine failure but caused a loss of performance.

**Figure 10:**
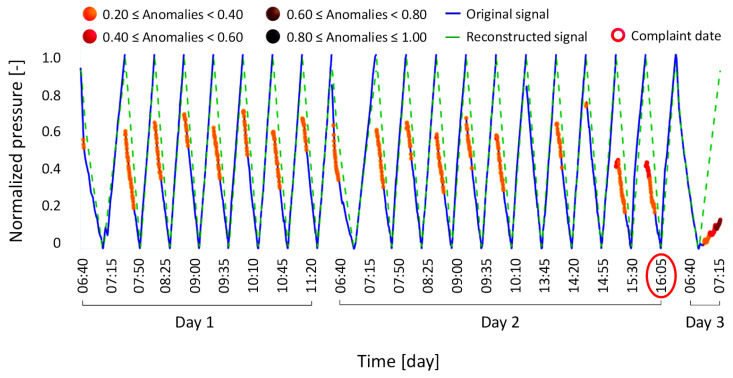
Results of the anomaly condition Type 4, where issues with the conductivity sensor and drift in the weight loss sensor did not cause machine failure but resulted in a loss of performance.

**Figure 11:**
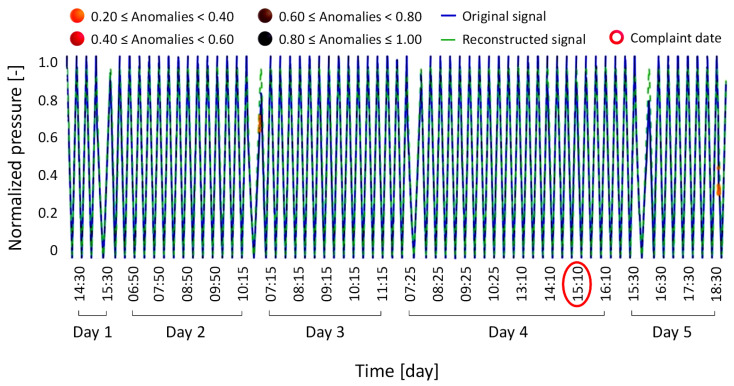
Results of the anomaly condition Type 5, where an out-of-range arterial pressure caused a machine failure, which in fact was not revealed by the model as a problem related to the weight loss sensor.

**Figure 12:**
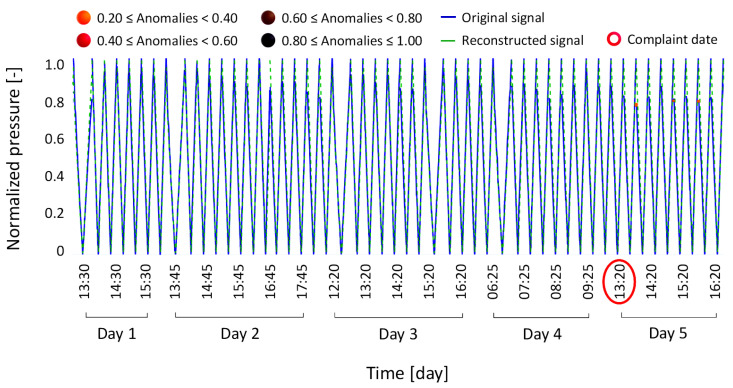
Results of the anomaly condition Type 6, where the presence of air in the extracorporeal blood circuit caused a machine failure, which in fact was not revealed by the model as a problem related to the weight loss sensor.

**Figure 13:**
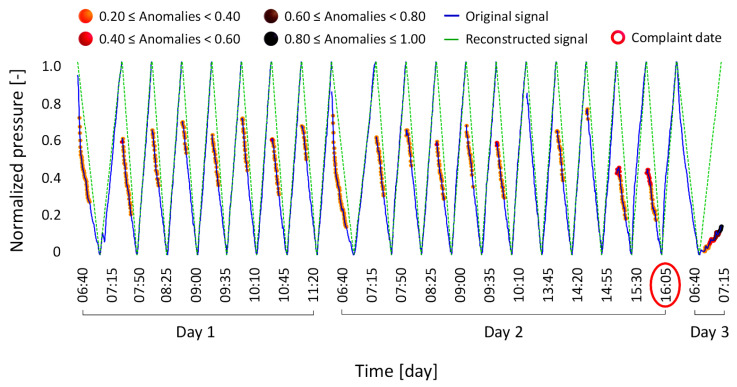
Results of the linear regression model for the case involving a conductivity sensor problem and drift in the weight loss sensor, which did not cause machine failure but resulted in a loss of performance (anomaly condition Type 4, previously analyzed in Figure 10 with the LSTM model).

Figure 7 shows the reconstructed signal (dashed green line) for anomaly condition Type 1, overlapped with the original signal (solid blue line). Machine failure events are highlighted with red circles along the abscissa-axis, which represents time in days, further divided into hourly intervals. The same legend is consistently used in the subsequent figures (Figure 8, Figure 9, Figure 10, Figure 11, Figure 12 and Figure 13).

The model-reconstructed signal confirmed the failure of the weight loss sensor self-test, as indicated by the brown and black markers. Notably, the model detected signs of a potential failure three days in advance, as shown by the orange, red, and brown markers highlighting anomalous points. Furthermore, following the recalibration attempt, the model identified another component malfunction, indicating an increased risk of a subsequent failure, which indeed occurred just four days after the initial machine breakdown. This first case thus demonstrates the model’s ability to detect early signs of failure, an aspect that could have supported a timely replacement of the component and, potentially, prevent the second breakdown. The magnitude distribution of the anomaly defined in the anomaly condition Type 1, ranging from 0.08 to 1 and categorized by color bands, was adopted as the reference framework for all subsequent cases. Based on these results, and following consultations with technical experts in component drift, an interpretative scheme was developed to associate each color band with a corresponding level of machine failure severity. The goal was to make the color legend meaningful and accessible to future users of the model, enabling them to interpret the results intuitively.

For the sake of brevity, and given the limited additional insight gained from repeating the absolute errors histograms for each case, these plots will not be reported again in the following sections. The focus will be on visualizing anomalies directly on the time-series signals, which provides a clearer understanding of the nature of each complaint.

#### 3.1.2. Failure Related to the Drift or Malfunction of Other Components

Anomaly condition Type 2, also involving a failure in the weight-loss sensor self-tests, regards an anomaly not caused by the drift of the sensor itself, but rather by the drift or malfunction of other components, which in turn affects the variables used in the sensor self-tests. Figure 8 shows the time-series signals corresponding to this condition.

The model properly detected the failure event on the day it occurred, as indicated by the brown and black marked dots. However, unlike the previous anomaly condition Type 1, the model did not predict the failure in the days preceding the event, since the few small anomalies observed before the event were not significant enough to justify the sudden test failure that occurred. This outcome is entirely reasonable, as the model was specifically trained to monitor the weight loss sensor and not to generalize failures unrelated to this component. Although this case does not demonstrate the preventive role of the model, it confirms that the model operates reliably within its intended scope, which is to alert operators only when failures directly affect the weight loss sensor.

### 3.2. Performance Loss Related to Partial Drift of the Weight Loss Sensor

Anomaly condition Type 3 involves a complaint regarding potential weight loss sensor drifts that did not cause failures of the machine but were associated with a loss of performance reported by the hospital operators.

Figure 9 illustrates the corresponding time-series signals.

The model-reconstructed signal confirmed the presence of a drift in the weight loss sensor on the day the complaint was filed, successfully detecting the anomaly up to five days before it was reported by the hospital.

This scenario exemplifies the exact application for which the model was designed. By detecting and reporting such anomalies in advance, the model can help operators take timely action, prompting technical intervention before a critical failure occurs. This approach would significantly reduce unplanned downtime and operational interruptions caused by emergency calls and delayed treatment, ultimately contributing to more efficient and proactive system maintenance.

Anomaly condition Type 4 involves a complaint initially associated with a potential malfunction of the conductivity sensor, which was causing a loss of performance in the dialysis machine. During the investigation, an ongoing drift in the weight loss sensor was also identified, as shown in Figure 10.

The conductivity sensor is a component responsible for measuring the concentration of electrolytes in the dialysis fluid, ensuring that the dialysate composition remains within safe and prescribed conductivity levels. Following technical analysis, the issue was identified as caused by the presence of air in the hydraulic circuit. Once the necessary corrective actions were taken to solve the problem, it was considered that the same cause might have also affected the weight loss sensor. This hypothesis was later confirmed.

Considering the date of the conductivity sensor complaint, the reconstructed signal reveals that, during the same time window, the weight loss sensor also exhibited signs of degraded performance. This is clearly indicated by the orange and red anomaly markers, which culminate in brown markers on the last available day of data for this case.

Although the reported issue concerned the conductivity sensor, the model would have enabled early detection of a concurrent drift in the weight loss sensor caused by the same underlying factor, which otherwise went unnoticed. In particular, this case shows that the model can identify weight loss sensor drifts even when the reported issue is apparently unrelated and involves other components, thus confirming its robustness.

### 3.3. Failure Unrelated to Weight Loss Sensor or Interfering Components

To further validate the reliability and specificity of the model, anomaly condition Type 5 illustrates additional tests conducted on signals associated with complaints unrelated to the weight loss sensor and not expected to affect its behavior or that of the pressure sensor used in its sensor self-tests. Figure 11 presents the results for this scenario.

In particular, the model was tested using weight loss sensor calibration signals extracted from a complaint regarding a machine shutdown caused by elevated pre-filter arterial pressure. Pre-filter arterial pressure refers to the blood pressure measured before the dialyzer module, and an elevated value may indicate vascular access problems, such as needle obstruction or restricted blood flow. However, this type of issue does not affect the calibration process or the proper functioning of the weight loss sensor. Given this context, the expected outcome for the model was a signal reconstruction free of anomalies, reflecting a properly functioning weight loss sensor. As shown in Figure 11, the model’s output met this expectation, with no significant anomalies detected through the signal.

This result demonstrates the model’s ability to accurately reflect the true behavior of the weight loss sensor, thereby minimizing false positives caused by unrelated issues.

Figure 12 illustrates a similar validation case, identified as anomaly condition Type 6. This instance involves a complaint triggered by an alarm from the air detector in the extracorporeal blood circuit, which was later confirmed to be caused by the actual presence of air in the circuit. Since this issue does not affect the hydraulic circuit and therefore does not influence the weight loss sensor, the self-test signals of the weight loss sensor were expected to remain unaffected. The model confirmed this expectation by detecting no abnormalities in the signals.

### 3.4. Conclusions of the LSTM Model Results

Based on the results presented in Section 3.1, Section 3.2 and Section 3.3, Table 2 associates the magnitude of detected anomalies to potential issues, categorized by severity.

**Table 2:** Classification of anomaly magnitudes for early detection. The concept of anomaly magnitude and the definition of the reference ranges are illustrated in Figure 6.

Anomaly Magnitude (AM)	Severity of the Issue
0.08 ≤ AM < 0.20	Slight anomalies due to minor external disturbances and system inaccuracies
0.20 ≤ AM < 0.40	More significant anomalies due to a small component drift
0.40 ≤ AM < 0.60	Very significant anomalies due to a significant component drift, indicative of possible upcoming machine failures
0.60 ≤ AM < 0.80	Serious anomalies indicating an impending machine failure
0.80 ≤ AM ≤ 1.00	Very serious anomalies indicative of an ongoing machine failure

Furthermore, the classification presented above highlights the model’s potential to prioritize anomalies based on their severity, assisting clinicians in planning preventive maintenance to avoid machine failures and, consequently, minimize downtime. This approach not only facilitates faster decision making but also enables targeted interventions to reduce risk.

### 3.5. Regression Linear Model

Alongside the LSTM approach, a simple linear regression model was developed to approximate the average trend of the pressure–time signal observed during the sensor self-tests [31]. The model was theoretically defined to reproduce the typical “V-shape” of the signal by enforcing a two-segment structure: a descending linear slope from 1 to 0, followed by an ascending slope from 0 to 1. These segments were constructed by considering the specific duration of the sensor self-tests, ensuring that the transition point between the two slopes aligned the expected inflection in the signal shape.

The performance of the linear regression model was evaluated on the same dataset as the LSTM model, enabling a direct and fair comparison. The analysis focused on key factors such as sensitivity to signal drift, ease of use, and model generalizability.

Although the linear regression approach was straightforward and easy to interpret, it showed clear limitations in detecting subtle deviations in the signal. In most test cases, the linear model yielded results consistent with those of the LSTM, confirming its reliability under standard conditions. In order to avoid redundancy, the majority of these results are not reported. However, an exception was observed in anomaly condition Type 4, which involved a conductivity sensor issue accompanied by a concurrent drift in the weight loss sensor. This resulted in a performance decline without causing machine failure.

In this specific case, the LSTM model correctly reconstructed the signal and did not flag it as a failure, aligning with the official diagnosis provided by technical staff following the hospital’s complaint. Conversely, the linear regression model produced a reconstruction that erroneously suggested a machine failure. This was indicated by the presence of a black anomaly marker in Figure 13 which, according to the severity classification established in Table 2, corresponds to a recent failure event.

Since no such failure was confirmed by the technicians, the output of the linear regression model in this case must be considered a false positive. This further confirms the LSTM model’s overall advantage in scenarios where subtle anomalies must be detected with high precision and reliability.

Furthermore, due to the presence of two types of sensor self-tests, one shorter and one longer, the duration and the inflection point between the descending and ascending segments vary accordingly. As a result, it was necessary to implement two separate models for the linear regression approach, each specifically tailored to the distinct duration of one of the sensor self-test types. On the other hand, the LSTM model did not require this distinction. Trained on complete sequences of both types of sensor self-test, it was able to naturally learn the timing differences between them without the need for separate configurations or manual intervention. This highlighted the practical advantages of the LSTM approach, which can be uniformly applied to similar signal types that vary in duration or structure, without requiring the user to redesign or adapt the model for each specific case.

Furthermore, the linear regression approach is inherently rigid and tailored to a specific signal shape. When the morphology of the signal changes significantly, the linear model becomes ineffective. In contrast, the high level of generalizability of the LSTM allows the model to be retrained on signals with entirely different shapes and dynamics, potentially achieving strong performance without any changes to the model structure [32].

This aspect, along with the others previously discussed, highlights that although the linear regression model is quite effective for this specific type of signal, the flexibility, accuracy, and generalizability of the LSTM in handling time-dependent sequences make it a more powerful solution compared to alternative non-AI approaches.

## 4. Conclusions

In this study, an AI-based predictive maintenance tool for dialysis machine components, with a focus on detecting weight loss sensor drift, was developed. By comparing a traditional linear regression model with an LSTM neural network, the superior diagnostic capability of the LSTM approach was demonstrated. Specifically, the LSTM model was trained on pressure–time signals recorded during routine sensor self-tests under normal operating conditions, learning to reconstruct the characteristic signal shape associated with properly functioning components, with a maximum reconstruction error of 0.08. Deviations over this value between the reconstructed signals and unseen data were then used to detect anomalies, indicating either sensor drift or broader system malfunctions.

A key strength of this study lies in the real-world validation of the model. It was tested on actual complaints reported by hospitals, where the causes of drifts or malfunctions had previously been identified and confirmed by technical personnel. In these cases, the model’s anomaly predictions aligned with the outcomes of post-event analyses, confirming its diagnostic reliability. Furthermore, in several instances, the model successfully anticipated issues up to five days in advance, by identifying subtle sensor degradations before they escalated to trigger alarms or disrupt treatment. These early warnings demonstrated the model’s capability to support routine calibration processes by detecting sensor drift between scheduled tests.

The model also demonstrated robustness and specificity by successfully distinguishing failures caused by the weight loss sensor from those originating in other components. This selective sensitivity offers valuable diagnostic guidance, enabling technicians to isolate root causes more efficiently and avoid unnecessary component replacements. Even in cases where no anomalies were detected prior to a failure, the “clean” output of the model provided valuable insight by redirecting investigations toward alternative sources, thereby supporting more accurate and efficient troubleshooting.

Although the calibration signals analyzed in this study are relatively regular and could be theoretically interpreted using simpler methods, such as the linear regression model (as compared herein), the use of an LSTM network has proved to be decisively advantageous. Unlike linear models, which rely on manual feature selection and impose strong assumptions about the data, LSTMs automatically learn complex temporal dependencies from sequential inputs, enabling greater adaptability across a wide range of operating conditions and signal types. This scalability and generalization capability make LSTM-based methods particularly well-suited for predictive maintenance in dynamic, data-rich environments, where they can be continuously re-adapted through retraining.

In addition to optimizing maintenance strategies and minimizing unplanned downtimes, this approach has the potential to significantly enhance equipment availability and patient safety in dialysis treatments. Its future integration into home dialysis systems, where technical supervision is limited, could further amplify its impact by enabling real-time monitoring and automated support.

Future work may explore real-time deployment on dialysis platforms, validation of additional models, extension to other sensors, and integration into remote monitoring systems. Furthermore, refining the anomaly classification framework by moving beyond the fixed rule-based threshold adopted in this study toward adaptive thresholds could enhance both the flexibility of the approach and its clinical relevance.

Since the proposed LSTM model was developed as an unsupervised reconstruction-based approach, another limitation is the absence of ground-truth anomaly labels in the dataset. As a result, conventional supervised evaluation metrics could not be applied. Model validation was instead based on real-world complaint cases confirmed through technical inspections. Future work should focus on collecting annotated and more extensive datasets, which would enable the use of standard statistical metrics and support a more systematic assessment of model performance. All together, these advances could drive the evolution toward smarter, more resilient, and sustainable healthcare systems powered by artificial intelligence, aligning with the broader goals of digital transformation in medical technology.

## Figures and Tables

**Table 1:** Cases analyzed with the LSTM model, categorized by different complaint reasons.

Anomaly Condition	General Reason for the Complaint	Analyzed Cases	Detailed Reason for the Complaint
Type 1	Machine out of order due to weight loss sensor self-test failure	2	Failure related to weight loss sensor drift
Type 2	Machine out of order due to weight loss sensor self-test failure	2	Failure related to the drift or malfunction of other components whose behavior affects the variables involved in the weight loss sensor self-tests
Type 3 Type 4	Machine not out of order but with performance loss in the weight loss sensor	4	Performance loss related to partial drift of the weight loss sensor
Type 5 Type 6	Machine out of order not due to weight loss sensor self-test failure	4	Failure not related to weight loss sensor drift or other components whose malfunctioning may affect the weight loss sensor

## Data Availability

The original contributions presented in this study are included in the article. Further inquiries can be directed to the corresponding author.
